# An omics review and perspective of researches on intrahepatic cholestasis of pregnancy

**DOI:** 10.3389/fendo.2023.1267195

**Published:** 2024-01-08

**Authors:** Min Wang, Lingyan Chen, Jingyang Li, Yilan You, Zhiwen Qian, Jiayu Liu, Ying Jiang, Tao Zhou, Ying Gu, Yan Zhang

**Affiliations:** ^1^ Center for Reproductive Medicine, The Affiliated Wuxi Maternity and Child Health Care Hospital of Nanjing Medical University, Wuxi, China; ^2^ Department of Gynaecology and Obstetrics, The Affiliated Wuxi Maternity and Child Health Care Hospital of Nanjing Medical University, Wuxi, China; ^3^ Wuxi Maternity and Child Health Care Hospital, Wuxi School of Medicine, Jiangnan University, Wuxi, China

**Keywords:** genomics, intrahepatic cholestasis of pregnancy, metabolomics, transcriptomics, perinatal complication, precise diagnosis, proteomics

## Abstract

Intrahepatic cholestasis of pregnancy (ICP) is one of the common pregnancy complications that may threaten the health of both pregnant women and their fetuses. Hence, it is of vital importance to identify key moleculars and the associated functional pathways of ICP, which will help us to better understand the pathological mechanisms as well as to develop precise clinical biomarkers. The emerging and developing of multiple omics approaches enable comprehensive studies of the genome, transcriptome, proteome and metabolome of clinical samples. The present review collected and summarized the omics based studies of ICP, aiming to provide an overview of the current progress, limitations and future directions. Briefly, these studies covered a broad range of research contents by the comparing of different experimental groups including ICP patients, ICP subtypes, ICP fetuses, ICP models and other complications. Correspondingly, the studied samples contain various types of clinical samples, *in vitro* cultured tissues, cell lines and the samples from animal models. According to the main research objectives, we further categorized these studies into two groups: pathogenesis and diagnosis analyses. The pathogenesis studies identified tens of functional pathways that may represent the key regulatory events for the occurrence, progression, treatment and fetal effects of ICP. On the other hand, the diagnosis studies tested more than 40 potential models for the early-prediction, diagnosis, grading, prognosis or differential diagnosis of ICP. Apart from these achievements, we also evaluated the limitations of current studies, and emphasized that many aspects of clinical characteristics, sample processing, and analytical method can greatly affect the reliability and repeatability of omics results. Finally, we also pointed out several new directions for the omics based analyses of ICP and other perinatal associated conditions in the future.

## 1 Introduction

Intrahepatic cholestasis of pregnancy (ICP) is one of the common pregnancy complications. It is characterized by the symptoms of itching (pruritus), and can be confirmed by the detection of increased serum bile acids. The incidences of ICP are obviously various in different countries, which are range from less than 1% to more than 27% (1). It occurs more commonly in a few cold regions such as Finland and Chile, especially in winter months. The latest cohort shows that the overall incidence of ICP in Chinese population is about 6.06% (2). The risk factors associated with ICP include but not limited to advanced maternal age, multiple gestation, and multiplicity of pregnancy. Besides, several genetic mutations in MDR3 and MRP2 are also reported to be involved in the etiology of ICP (3).

ICP usually appears in the late second and early third trimesters of pregnancy (4). Although the clinical symptoms and biochemical abnormality of ICP resolve rapidly after delivery, it can be a serious threat to both maternal and fetal health. Besides the pruritus and impaired liver function, recent studies also showed that ICP is associated with increased risk of other pregnancy complications such as gestational diabetes mellitus (GDM) and preeclampsia (PE) (5, 6). Most importantly, it also increases the risk of adverse perinatal outcomes, including preterm delivery, meconium excretion into the amniotic fluid, respiratory distress syndrome, and sudden intrauterine fetal death (7). Hence, it is important to early and precisely diagnosis of ICP, which will benefit the treatments to prevent adverse effects on both pregnant women and their fetuses.

Although the complex pathogenesis is not fully understood, the estrogen-bile acid axis is known to play a key role in the development of ICP (8). The current laboratory tests of ICP are mainly based on the detection of serum bile acid and several liver functional enzymes (such as aspartate transaminase and alkaline phosphatase) (9). Over the past decades, the developing of multiple “omics” techniques provides comprehensive approaches to systematically screening novel targets for various prenatal related conditions (10). Compared to single gene or pathway based analysis, omics-driven study offers a global view of the pathological or physiological conditions at the level of different molecular systems.

**Figure 1:**
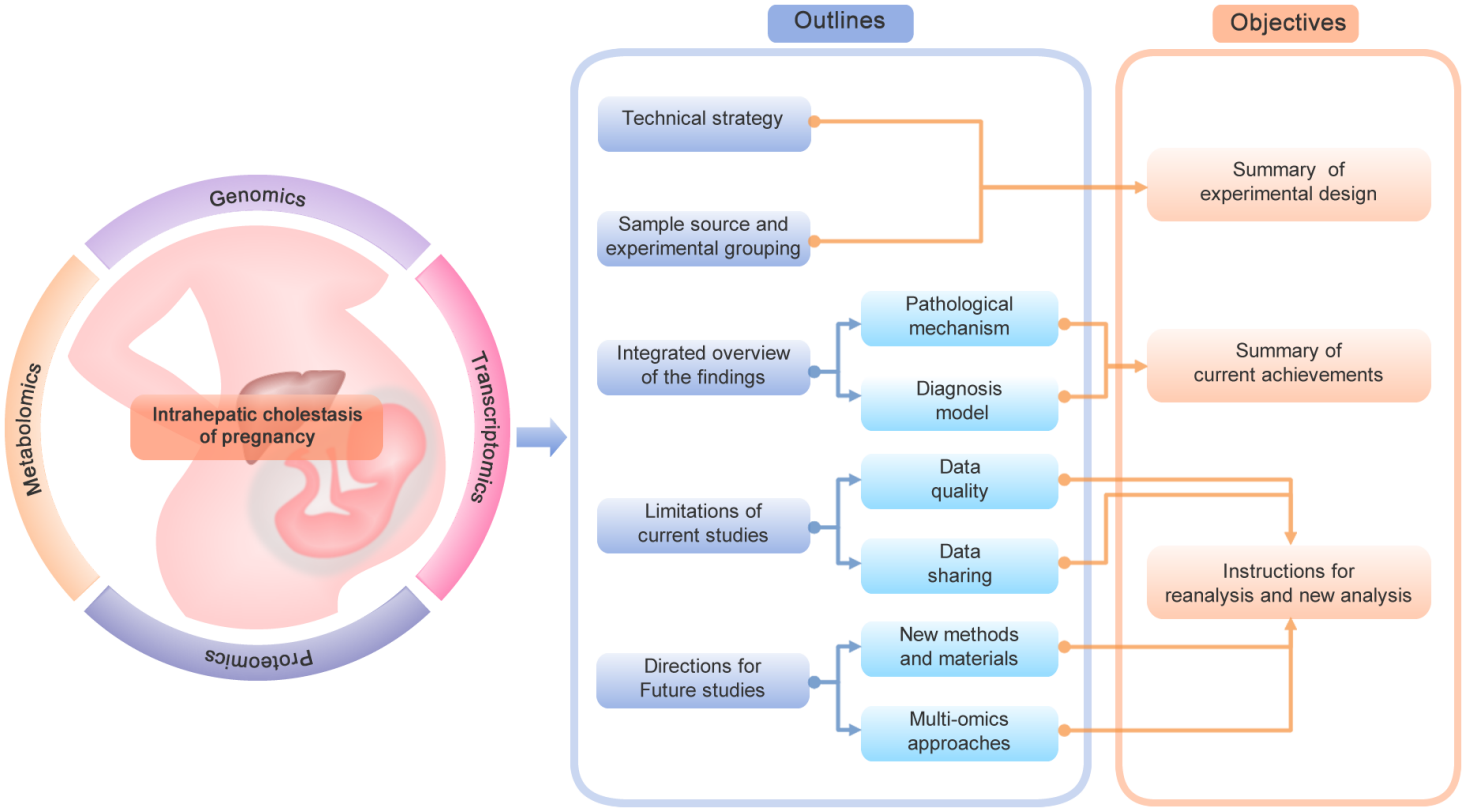
The framework of the current review. The diagram shows the main contents and objectives of the current review.

Several reviews and meta-analyses have already summarized recent knowledge about clinical symptoms, delivery risks, biochemical signatures, pharmacological treatments and management of ICP (3, 11, 12). We have summarized the canonical and novel biomarkers involved in the pathogenesis of ICP in a previous review (13). In addition to the dominant role of estrogen-bile acid axis, we also illustrated the role of genetic factors, hormones, hypoxia, and inflammatory factors in the regulation of ICP. However, more and more omics based studies of ICP have been performed to screen for novel targets or pathways in recent years. It is in urgent need to comprehensively collect and summarize these datasets to obtain an overview of the current progress, limitations and future directions of the omics based studies of ICP. The correct selection of technical strategy, sample type and sample group is the first and key step of omics studies to address a specific clinical issue. Thus, in the present review, we will first summarize the technical strategy, sample source and experimental grouping of these studies, aiming to provide a connection between the experimental design and the corresponding research objective (**Figure 1**). Second, since omics technologies serve as both discovery and translational tools for clinical researches. We will then integrate and summarize the main findings according to the two corresponding categories: pathological mechanism and diagnosis model, which can offer an overview of the current achievements. Finally, to provide constructive instructions for possible reanalysis and new analysis in the future, we will also evaluate the limitations of current studies and propose new directions for future studies. Although the present review is a broad review without in-depth meta-analysis and re-analysis, it will provide well-organized information about the experimental design and main findings of these studies as **Supplementary Tables**.

## 2 Classification of omics studies by technical strategy

**Figure 2:**
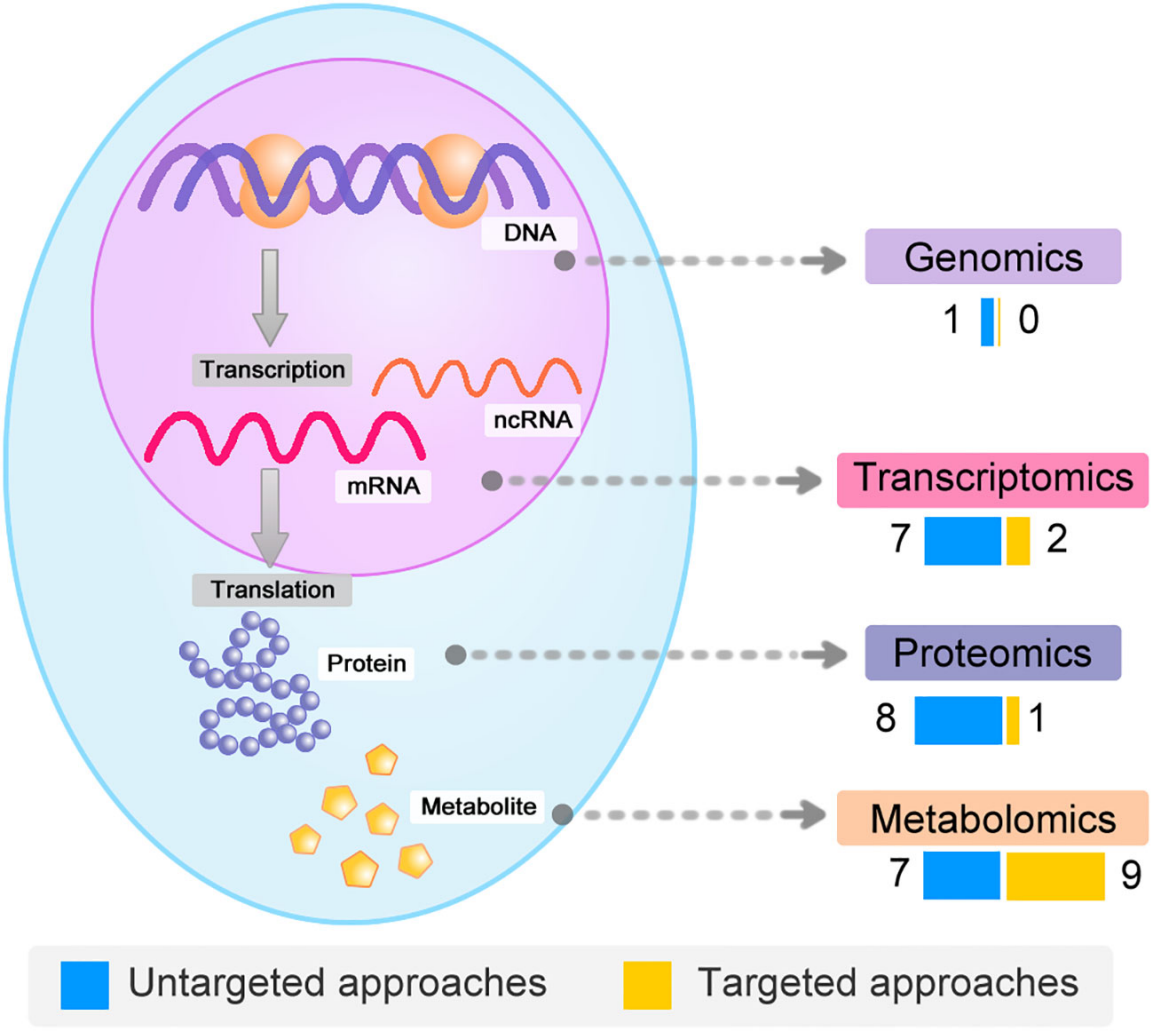
Global overview of the omics datasets. Global overview and classification of omics datasets of ICP according to the molecular types.

A comprehensive literature search of PubMed database was conducted by using the combined keywords of the disease term (“Intrahepatic Cholestasis of Pregnancy”) and one of the technology term (“genomics”, “transcriptomics”, “proteomics”, or “ metabolomics”) separately. We further manually curated the results to filter non-English articles and those studies without original datasets. After the extensive review, we obtained a list of 35 omics based studies of ICP (**Supplementary Data 1**). In addition to different omics types, we also divided these studies into two main catalogues according to the theoretical coverage: untargeted and targeted (**Figure 2**). The untargeted studies are intended to investigate all the possible moleculars in the samples, while the targeted studies aim to test only a specifically selected list of molecular targets. Generally, the untargeted methods for genomics and transcriptomics are based on next-generation sequencing and microarray. And mass spectrometry is the common technology for untargeted and targeted proteomics and metabolomics. We also incorporated gene or protein panel studies based on traditional low-throughput methods to obtain a full overview of the research field. This section is mainly focus on the classification and features of different omics strategies, while the summary of the corresponding experimental design and findings are described in the following sections.

### 2.1 Genomics strategies

Based on the central dogma, DNA sequence is the initial and core component of multiple omics. It contains both coding and non-coding information for the following RNA transcription and protein translation. Although the whole sequences of human genome are completely sequenced, genomics approaches are widely used to decode disease associated gene mutations and variations among individuals. In addition to the changes of DNA sequence itself, epigenomics approaches aim to uncover DNA modifications or chromatin states that could be dysregulated to cause disease indirectly. Currently, there lacks of direct genomics and epigenomics analyses of ICP. A recent study carried out the first genome-wide association studies (GWAS) and meta-analyses of ICP by using three large cohorts of whole-genome sequence (WGS) data: the NIHR-RD, 100KGP and FinnGen (14). A total of 11 genomic loci were found to be significantly associated with ICP. And further functional prioritization showed that liver-enriched genes and liver-specific cis-regulatory elements may contribute to the susceptibility of ICP. Similar to other GWAS analyses of human diseases (15), most of the loci are non-coding and synonymous variants, with no direct relevance to ICP. Thus, further validation and experiments are needed to clarify the causality and downstream pathways.

### 2.2 Transcriptomics strategies

Transcriptomics focuses on the analysis of the expression levels and splicing patterns of both coding and non-coding RNAs. GWAS analysis only identifies DNA elements that may affect the regulation of gene expression among individuals, while transcriptomics are usually used to identify common transcripts that are differentially expressed between different samples. A total of 9 studies of transcriptomics analyses of ICP have been collected in this review (16–24), including 7 untargeted and 2 targeted studies (**Table 1**). Among the untargeted studies, mRNA, miRNA, and lncRNA microarrays are used to detect differentially expressed coding and non-coding genes. Compared to microarray strategies, the high-throughput RNA-sequencing technology could identify more genes, and is also capable of identifying alternative splicing. However, microarray-based transcriptomics are still useful and efficient in analyzing of clinical samples (25). Additionally, the remaining 2 targeted studies tested the selected gene expression profiles of mitochondrial or bile acid transport related genes by using the method of RT-PCR (reverse transcription-polymerase chain reaction).

**Table 1:** List of transcriptomics datasets.

Published year	Coverage	Research objective	Grouping design	Sample source	Sample type	Analytic method	Reference
2009	Targeted	Pathogenesis	ICP vs Control	Human body	Placenta	RT-PCR	(16)
2010	Untargeted	Pathogenesis	ICP vs Control	Human body	Placenta	Gene microarray	(17)
2010	Untargeted	Pathogenesis	Pregnant, Cholate-fed and Control (mouse model)	Mouse	Liver	Gene microarray	(18)
2013	Untargeted	Pathogenesis	ICP female offsprings vs Control female offsprings (mouse model)	Mouse	Liver	Gene microarray	(19)
2014	Untargeted	Pathogenesis	mild ICP vs severe ICP vs Control	Human body	Placenta	Gene microarray	(20)
2016	Targeted	Pathogenesis	ICP vs Control	Human body	Placenta	RT-PCR	(21)
2018	Untargeted	Diagnosis	ICP vs Control	Human body	Serum	miRNA microarray	(22)
2021	Untargeted	Diagnosis; Prognosis	ICP vs Control	Human body	Serum	lncRNA microarray	(23)
2022	Untargeted	Diagnosis	ICP vs Control	Human body	Serum	miRNA microarray	(24)

### 2.3 Proteomics strategies

Although transcriptomics approaches can be used to detect the expression of protein-coding genes. It is known that mRNA and the corresponding protein expressions are not well correlated (26). Thus, mass spectrometry based proteomics can be used to study the expression levels, isoforms and post-translational modifications of proteins. The present review collected a total of 9 proteomics studies of ICP (27–35), including 8 untargeted and 1 targeted studies (**Table 2**). There are three types of technologies for the analyses of untargeted proteomics studies, including: 2D-PAGE MS (two-dimensional polyacrylamide gel electrophoresis followed by mass spectrometry), DDA (data-dependent acquisition) based LC-MS/MS (Liquid chromatography–tandem mass spectrometry), and DIA (data-independent acquisition) based LC-MS/MS. These three strategies represent different developing stages of proteomics technologies (36). The 2D-PAGE MS relies on the selection of gel spots for the following MS detection, which is low-efficient and low-throughput. The DDA based LC-MS/MS is the most widely used high-throughput proteomics strategy to systematically analyze the protein composition. It can also be combined with various labeling methods, such as TMT (Tandem Mass Tag) and iTRAQ (Isobaric Tag for Relative and Absolute Quantitation), for the quantification of thousands of proteins in a single experiment. The recently emerged DIA based LC-MS/MS further improves the coverage and accuracy by scanning all the spectra in the sample, offering a comprehensive and unbiased approach for protein quantification. As a complement of MS based strategy, the target study is focused on the detection of mTOR signaling associated proteins by immunohistochemistry.

**Table 2:** List of proteomics datasets.

Published year	Coverage	Research objective	Grouping design	Sample source	Sample type	Analytic method	Reference
2013	Untargeted	Pathogenesis	ICP vs Control	Human body	Placenta	iTRAQ LC-MS/MS	(27)
2014	Untargeted	Pathogenesis	ICP vs Control	Human body	Placenta	2D-PAGE MS	(28)
2019	Untargeted	Pathogenesis	Taurocholate vs Control (*in vitro* cultured tissue model)	*In vitro* tissue	Placenta	iTRAQ LC-MS/MS	(29)
2019	Targeted	Pathogenesis	ICP vs Control	Human body	Placenta	Immunohistochemistry	(30)
2021	Untargeted	Diagnosis	ICP vs Control	Human body	Serum	DIA LC-MS/MS	(31)
2022	Untargeted	Pathogenesis	ICP vs Control	Human body	Placenta	DIA LC-MS/MS	(32)
2022	Untargeted	Pathogenesis	Chenodeoxycholic acid vs Deoxycholic acid vs Control	Cell line	HTR8 cells	TMT LC-MS/MS	(33)
2023	Untargeted	Pathogenesis	mild ICP vs severe ICP vs Control	Human body	Placenta	DIA LC-MS/MS	(34)
2023	Untargeted	Diagnosis	ICP vs Control	Human body	Plasma exosomes	DIA LC-MS/MS	(35)

### 2.4 Metabolomics strategies

Recently, a new theory has been proposed that metabolites play important roles in connecting the central dogma and the biological phenotype (37). Hence, metabolomics not only provide qualitative or quantitative information about various metabolites, it can also offer phenotype associated information about gene activities by pathway mapping. It has been a useful tool for discovering and developing biomarkers for various human diseases. The current review collected a total of 16 metabolomics studies of ICP (38–53), including 7 untargeted and 9 targeted studies (**Table 3**). Mass spectrometry is also the basic technology of metabolomics. However, compared to the detection of the intact metabolite ions (on the MS1 level), the application of further fragmentation (on the MS2 level) can greatly improve the recognition of metabolite structures (54). Additionally, it should also be noted that different separation and processing methods, such as gas (GC) or liquid (LC) chromatography, may be sensitive to a specific group of metabolites (55).

**Table 3:** List of metabolomics datasets.

Published year	Coverage	Research objective	Grouping design	Sample source	Sample type	Analytic method	Reference
2016	Targeted	Diagnosis	ICP vs GDM vs Control	Human body	Serum	LC-MS/MS	(38)
2017	Untargeted	Diagnosis	ICP vs Control	Human body	Urine	LC-MS	(39)
2018	Untargeted	Diagnosis	ICP vs Control	Human body	Hair	GC-MS	(40)
2018	Targeted	Diagnosis	mild ICP vs severe ICP vs Control	Human body	Urine	LC-MS/MS	(41)
2018	Targeted	Diagnosis	ICP vs Control	Human body	Serum	LC-MS/MS	(42)
2019	Targeted	Diagnosis	AHP vs ICP vs Control	Human body	Urine	LC-MS/MS	(43)
2019	Targeted	Pathogenesis	G60, G90, and L0	Pig	Serum	LC-MS/MS	(44)
2021	Untargeted	Diagnosis	ICP vs Control	Human body	Placenta	LC-MS	(45)
2021	Targeted	Diagnosis	mild ICP vs severe ICP vs Control	Human body	Plasma	LC-MS/MS	(46)
2021	Targeted	Diagnosis	ICP1 vs ICP2 vs ICP3 vs ICP4 vs Control	Human body	Serum	LC-MS/MS	(47)
2022	Untargeted	Pathogenesis	17α-Ethinyl estradiol, Paeoniflorin and Control	Rat	Serum; Faeces	LC-MS	(48)
2022	Untargeted	Pathogenesis	ICP vs Control	Human body	Plasma	LC-MS/MS	(49)
2022	Untargeted	Pathogenesis	mild ICP vs severe ICP vs Control	Human body	Plasma	LC-MS/MS	(50)
2022	Targeted	Diagnosis	AHP vs ICP vs Control	Human body	Serum	LC-MS/MS	(51)
2022	Targeted	Pathogenesis	Wt-Ve,WT-EE,TR-Ve,TR-EE ethinylestradiol (EE) administration to Mrp2-deficient (TR) rats and their wild-type (WT) controls	Rat	Plasma	LC-MS	(52)
2023	Untargeted	Pathogenesis	ICP vs Control	Human body	Placenta; Serum; Urine	LC–MS/MS	(53)

## 3 Summary of sample source and experimental design

**Figure 3:**
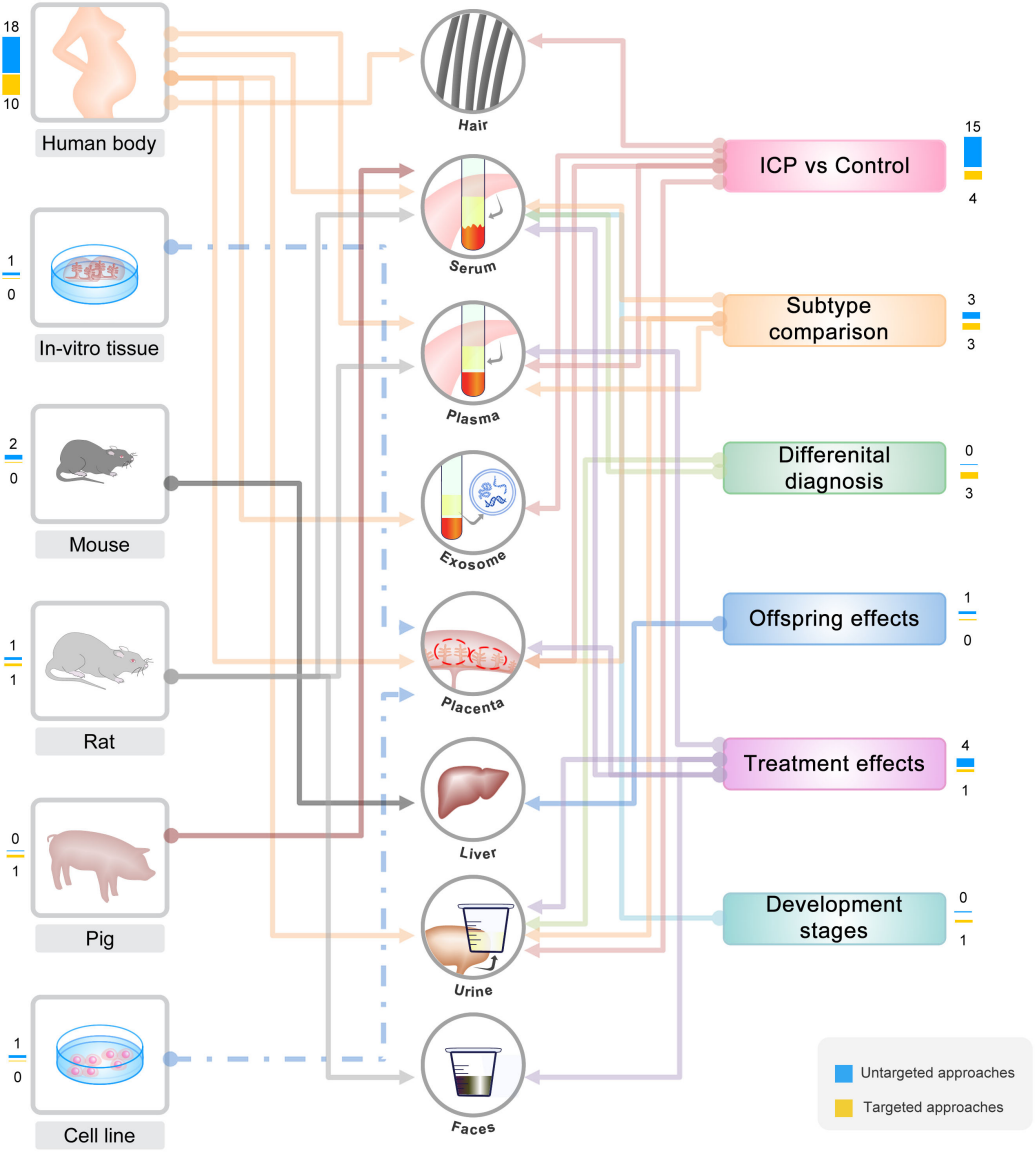
Summary and classification of the omics based studies. Summary and classification of omics based studies of ICP according to sample sources and experimental designs.

Sample selection determines the research objectives, and different sample types could reflect different aspects of a disease (**Figure 3**). GWAS datasets are based on the sequencing of blood sample, which is one of the most easily obtained clinical samples to reveal genome variations. For other omics datasets of ICP, most of the studies used patient-derived samples (including placenta, serum, plasma, urine, and hair), which are all non-invasively obtained due to the limitation of clinical sampling. However, several studies used other samples from *in vitro* tissues, cell lines or animal models, which greatly expand sample sources and improve the experimental designs. For example, the HTR8 cells were used to investigate the direct effects of BAs on placental trophoblast (33). The *in-vitro* cultured and treated placenta tissue was also used to investigate the direct effects of taurocholate on placental villous (29). In addition, the rat model was applied to explore the treatment mechanism of paeoniflorin by the analysis of serum and fecal samples (48). The mouse model was applied to explore the effects of ICP on female offsprings by the analysis of liver sample (19). Moreover, the pig model was applied to dynamically investigate the homeostasis of BA during the development of the fetus (44).

Most of the studies are based on the comparison of ICP patients and the pregnant women without common conditions. A few studies classified the ICP patients into mild and severe sub-groups, according to the serum total bile acid (TBA) level and clinical symptoms (20, 34, 41, 46, 50). One study further classified ICP patients into four subtypes based on three serum indicators: TBA, direct bilirubin (DBIL), and alanine aminotransferase (ALT) (47). In addition to the canonical cases of ICP, two studies also collected and investigated the cases of asymptomatic hypercholanemia of pregnancy (AHP), which is diagnosed only by the detection of elevated level of serum TBA without clinic symptoms (pruritus and jaundice) (43, 51). Moreover, one study also compared the serum BA profiles between ICP and gestational diabetes mellitus (GDM) for differential diagnosis (38).

## 4 Integrated overview of current omics studies

### 4.1 Identification of targets for exploring pathological mechanism

**Figure 4:**
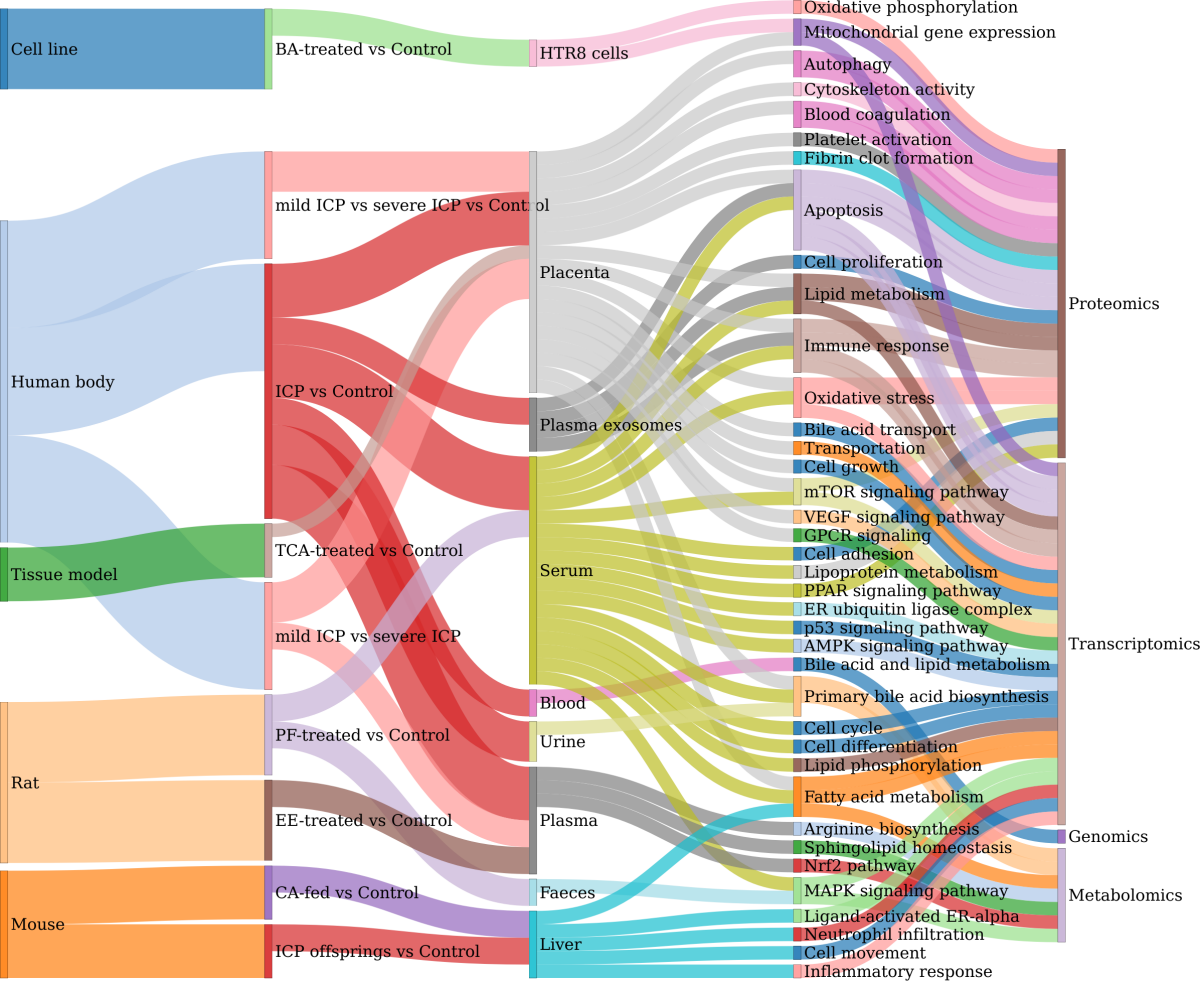
Summary of the functional pathways discovered in omics studies. Sankey plot for the relations between and among sample source, experimental comparison, sample type, functional pathways and omics types.

Next, we summarized and classified the main research objectives of these studies into two main groups: pathogenesis and diagnosis analyses. More specifically, the pathogenesis studies aim to identify key moleculars and pathways associated with the pathophysiology of ICP, its effects in the offsprings, or the treatment mechanism (**Supplementary Data 2**). Various functional terms and signaling pathways are found to be associated with ICP based on these omics studies, showing the complexity of the molecular mechanisms (**Figure 4**). A few common functional pathways (such as apoptosis, lipid metabolism, immune response, primary bile acid biosynthesis, and oxidative stress) are identified in multiple samples. Firstly, the results confirmed that the dominant regulatory pathway of estrogen- bile acid axis can be prioritized by omics studies. Secondly, it can be seen that many other pathways are not commonly found among different types of omics studies. This is mainly due to the differences of experimental grouping and sample types. Thus, these pathways may also represent different aspects of the regulations of ICP and its adverse outcomes. For example, the apoptosis index is found to be significantly higher in the placenta of ICP patients, compared to healthy pregnant women. And the key protein ERP29, which is known to induce apoptosis, is also verified to be over-expressed in the placenta of ICP group (27). Another example is that a trancriptomics analysis of placenta reveals a list of 8 hub regulatory genes (including CCL3, CCL25, CXCL6, CXCL14, CCR4, CCR6, CCR9 and IL-7R), which are involved in immune response. And further experiments of immune cell infiltration showed that the numbers of immune cells are increasing from mild ICP to severe ICP (20). However, it should be noted that many of the other functional pathways are only predicted by bioinformatics analysis. Thus, future experiments are needed to verify these pathways as well as to identify more key moleculars. Nevertheless, various types of omics studies has already provided a plenty of novel pathways and their targets for us to better understand the molecular mechanisms of the occurrence, progression, treatment and fetal effects of ICP.

### 4.2 Screening of biomarkers for developing diagnostic models

**Figure 5:**
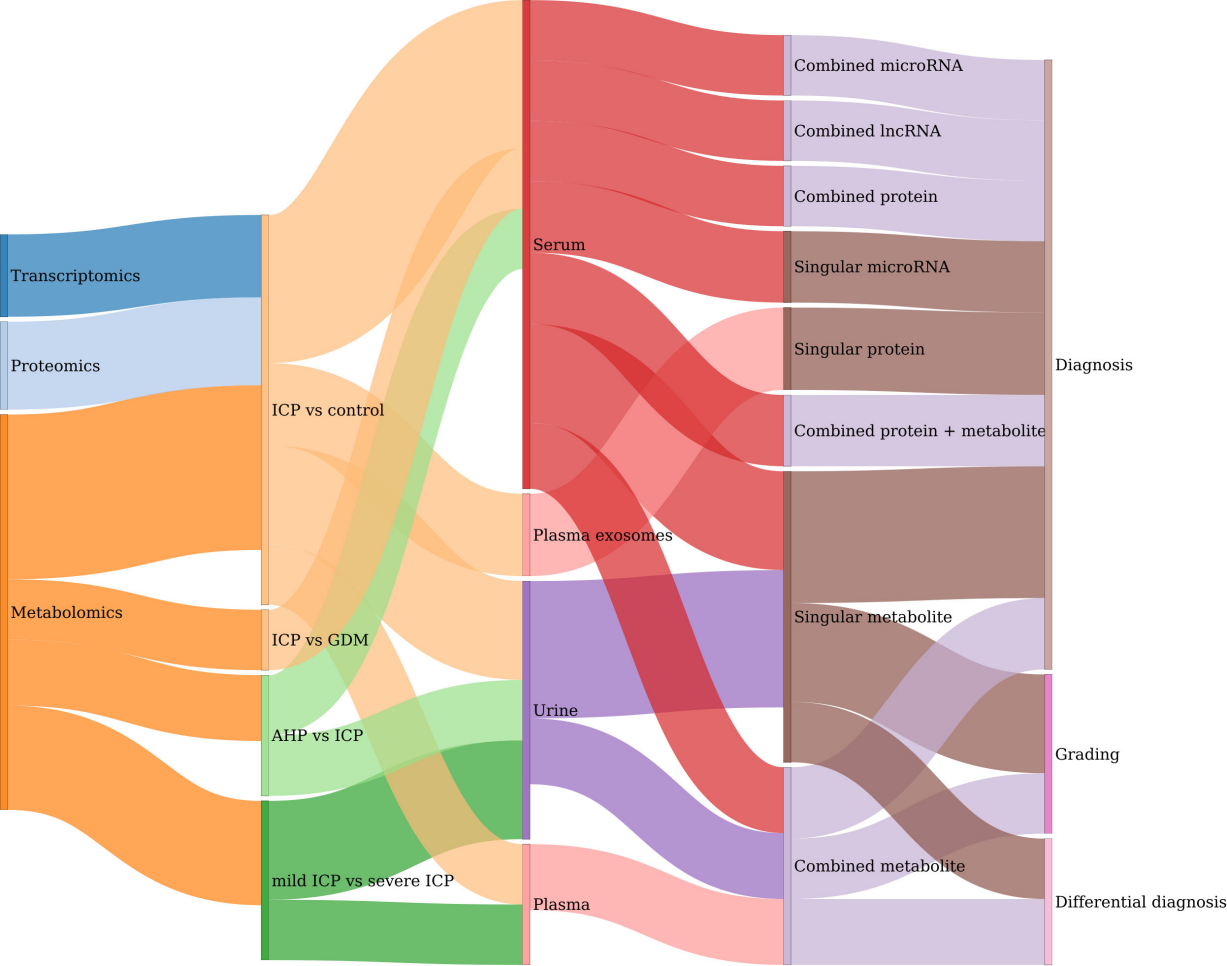
Summary of the diagnosis models developed in omics studies. Sankey plot for the relations between and among omics types, sample source, experimental comparison, molecular type and their clinical applications.

On the other hand, the diagnosis studies aim to screen for potential biomarkers for precision diagnosis, differential diagnosis and grading of ICP (**Figure 5**). By combing these studies, a total of 43 test models are obtained for the diagnosis of ICP and its related complications (**Supplementary Data 3**). Since omics-driven biomarker discovery usually requires multiple rounds of screening, the integrated **Supplementary Table** provides a useful resource for future evaluation and validation of these candidate biomarkers. Specifically, 31 testing models are used for the diagnosis of ICP, while 9 models are used for the grading of different types of ICP (mild versus severe). Additionally, there are also two and one models for differential diagnosis of AHP and GDM respectively. The biomarkers of these models are identified and tested in four types of samples: serum, plasma, plasma exosomes, and urine. Serum and plasma are the most commonly used body fluids for clinical diagnosis. Recently, exosomes derived from multiple tissues are found to be an ideal source of biomarkers due to its stability and functional connections (56). For example, five serum-derived exosomal proteins (including Elongation factor 1-alpha 1, Beta-2-glycoprotein I, Zinc finger protein 238, CP protein and Ficolin-3) are found to be promising biomarkers for the diagnosis of ICP (35). Finally, urine could used be a non-invasive source of biomarkers. For example, several sulfated bile acids (such as sulfated dihydroxy glycine bile acid, glycine cholic acid 3-sulfate, sulfated dihydroxy taurine bile acid and taurine cholic acid 3-sulfate) in urine can be used as potential biomarkers for the grading of mild and severe types of ICP (41).

Compared to singular molecular based model, the combination of multiple biomarkers can usually increase the diagnostic performance. For example, a panel with four serum proteins (including S100A9, LDHA, APOA1 and CHE) outperformed any of the individual indicators (31). Moreover, the combination of different types of moleculars can also greatly optimize the diagnostic efficiency. For example, the area under the curve (AUC) of an integrated model with two metabolites and one protein can be increased to 0.932 for early diagnosis of ICP by using the serum samples obtained from the first trimester (45). Among the models with outstanding discrimination (AUC>0.9), it can be seen that metabolites, especially various subtypes or derivatives of bile acids, are generally outperformed than proteins and other types of biomarkers. This may particularly due to the current diagnosis criteria, which is mainly based on the serum levels of BAs. However, the seeking of non-metabolite biomarkers may be helpful for the precise diagnosis and prognosis of ICP and its conditions in addition to metabolites. It may also be used to screen for key genes and regulatory pathways associated with the development of ICP. For example, several miRNAs are found to be potential diagnosis biomarkers for ICP. And functional prediction analyses further showed that these miRNAs could be the upstream regulators for fatty acid biosynthesis and mTOR signaling pathway (24).

## 5 Limitations of current omics studies

We previously proposed a refined framework of precision and translational proteomics for clinical studies with constructive suggestions (57). This framework could also be applicable for other omics studies to evaluate the data quality. Firstly, we suggest that it is needed to clearly describe the detailed experimental information. The minimal required and important information should include multiple aspects of clinical features, sample processing, and analytical method. Secondly, it is recommended to freely share the main results as well as the raw data, which can provide valuable resources for follow-up analysis and reanalysis. Thirdly, it is also of vital importance to perform additional verification experiments for initial screening analysis, in order to validate the results as well as to further identify reliable targets. Considering the above issues, we systematically assessed the information related to the experimental design, data availability and experimental verification of these omics datasets (**Supplementary Data 1**).

### 5.1 Experimental design

**Figure 6:**
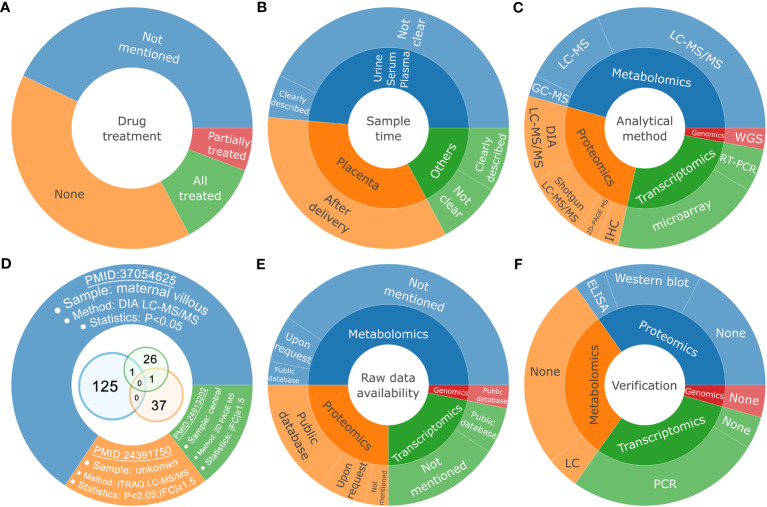
The limitations of the current datasets. Pie and venn diagrams for the differential classification of the experimental information associated with drug treatment **(A)**, sample time **(B)**, analytical method **(C)**, representative placenta proteomics datasets **(D)**, raw data availability **(E)** and experimental verification for untargeted studies **(F)**.

Since ICP threatens the health of both the pregnant women and the fetuses, it is usually treated with therapeutic drug such as ursodeoxycholic acid (UDCA) after diagnosis. Thus, it should be noted that treated-ICP is clearly not equal to untreated-ICP, considering both of the phenotypes and the molecular changes. However, only 14 studies (about 40%) collected the samples under the condition of none drug treatment (**Figure 6A**). A total of 6 studies used the samples treated or partially treated with drugs. These studies may produce ambiguous results to explain the intrinsic pathogenesis of ICP. Additionally, pregnancy is a long-term process, and can be divided into three trimesters. Hence, sample timing is also important to the interpretation and application of the omics results. For example, samples from the first trimester can be used for early predicting or exploring the initial etiology of ICP. The sampling time of placenta is generally known to be postpartum. However, most of the studies of serum, plasma and urine samples did not clearly mention the sampling time, making it hard to evaluate the potential usage of the results (**Figure 6B**). The placenta tissue is the maternal-fetal interface that connects the health of the mother and the child. The sampling position of placenta is of vital importance for interpreting the results, due to the complexity of the structure and composition of placenta (58). However, there is also no consistent standards and procedures of sampling among the 12 studies of placenta. Finally, the analytical and statistical methods for the initial screening of candidate markers are also varied among different studies (**Figure 6C**). As the evolving of omics based technologies, the newly emerged methods usually outperform than the old ones greatly. For example, the shotgun proteomics based on LC-MS/MS can identify a lot more proteins, compared to the low throughput and time-consuming approach based on 2D-PAGE. And the recently developed DIA proteomics further improves the quantitative accuracy and may be used directly for clinical diagnosis (59). Considering the above issues, the differences of these experimental information could make it hard to directly compare and integrate the results, even for the sample type of tissue. For example, we compared the differentially expressed proteins among and between three groups of proteomics analysis of placenta, and found that there are few overlapped proteins (**Figure 6D**). These protein groups are identified through different sampling position, proteomics method, and statistical criteria. Thus, it is still worth to perform independent experiments with the consideration of the above issues to obtain more reliable and repeatable results in the future.

### 5.2 Data availability

One of the benefits of omics-based study is that it could provide abundant resources for the academic community. Thus, to enable in-depth integration or reanalysis of the source datasets, it is encouraged to deposit the raw data to public databases. There are already many specialized data centers for the storage of different types of large omics data. For example, the Sequence Read Archive (SRA) is developed to store genomics or transcriptomics data based on next generation sequencing (60). The ProteomeXchange consortium provides standardize platforms for data submission and sharing (61). And the MetaboLights repository aims to store metadata and raw data associated with metabolomics experiments (62). However, only a few of these omics studies have uploaded the raw data to public databases (**Figure 6E**).

### 5.3 Experimental verification

Omics based initial studies (untargeted) usually generate a list of candidate moleculars ranging from tens to thousands (63). However, false positive remains a common problem due to complex factors, including but not limited to sample variation, sample quality, operational error, technical bias, and statistical limitation. Thus, it is also required to perform additional verification experiments to further check the quality of the results as well as to identify key targets. However, only less than half of the untargeted studies have performed verification experiments by using new methods and new samples (**Figure 6F**).

## 6 Suggestions of new research directions for future omics studies

**Figure 7:**
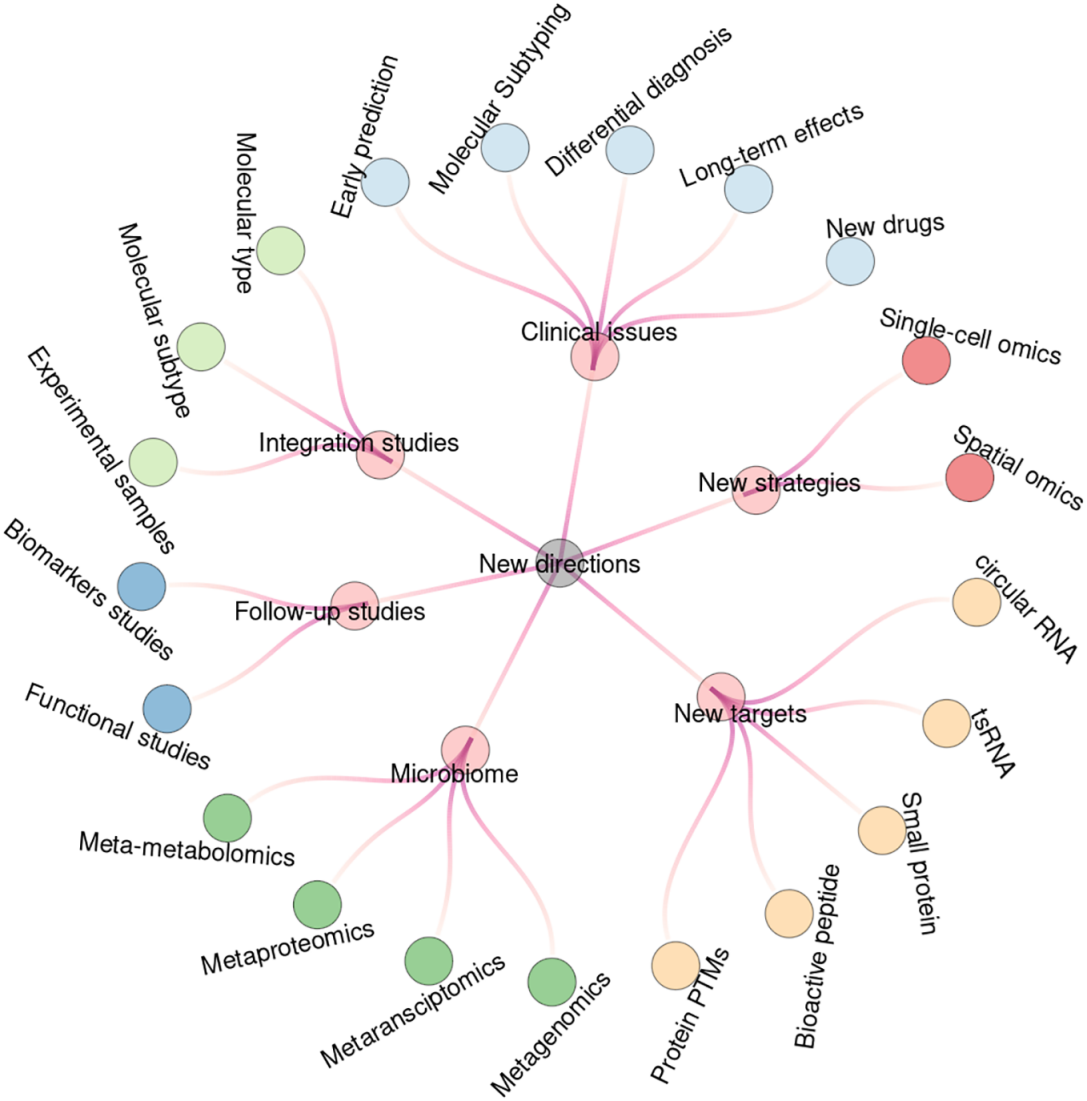
New research directions for future studies. Circular tree diagram shows the six groups and twenty-one subcategories of the new research directions for future studying of ICP based on omics strategies.

The current collected omics driven studies have already covered a broad range of research topics in the field of ICP. However, there are still many novel issues that could be worthy of attention in the near future. We divide the new directions into the following six categories (**Figure 7**). Some of the topics may have already been well studied in other pregnant complications such as GDM and PE. However, most of the issues could provide new directions or hints for the analyses of ICP and other pregnant complications by using various omics strategies.

### 6.1 New considerations for clinical issues

As mentioned above, most of the experimental designs focused on the comparison of ICP and the normal pregnant groups. However, ICP may threaten the health of both pregnant women and the fetus. Therefore, more attentions should be paid to the early prediction, precision diagnosis, differential diagnosis, birth outcome, long-term health effects and new treatments of ICP. Firstly, it was reported that a panel of four serum biomarkers could predict the risk of GDM in about 7 years before pregnancy (64). Thus, it would be interesting to find if there are such early biomarkers for ICP. Secondly, the current standards for diagnosis and treatment of ICP are quite simple without an international consensus (65). A few studies have classified ICP into different subtypes based on the symptoms, and found that their perinatal outcomes are different correspondingly (47). In respect of diagnosis period, ICP can also be divided into early-onset or late-onset groups (66). We then believe that omics-driven studies can further improve the precise classification of ICP in the future. Thirdly, as ICP is a pregnancy-associated hepatic disorder, it should be also comparatively analyzed with other liver diseases or conditions with similar symptoms (such as acute fatty liver, viral hepatitis, steatohepatitis, septic pyelonephritis, hyperbilirubinemia syndromes, bile duct obstruction and drug associated icterus) at the molecular level. Fourthly, it is also important to search for possible biomarkers for the evaluation of adverse birth outcome, including preterm labor, stillbirth, fetal distress, and low birth weight. Additionally, it is needed to investigate the long-term effects of ICP on both pregnant women and the fetuses. For example, the recurrence rate of ICP in subsequent pregnancies is high and the symptom tends to be more severe (65). And both the male and female children of ICP mothers may have high risk of metabolic diseases with altered lipid profiles (19). Finally, the current first-line therapy drug for ICP is ursodeoxycholic acid (UDCA) (67). There are also other potential drugs, which are also found to be complements or alternatives of UDCA for the treatment of ICP (12, 68). For example, the combination of rifampicin and UDCA is reported to be effective in the treating of severe ICP patients who are not sensitive to UDCA (69).

### 6.2 New strategies based on the state-of-the-art technologies

Traditional omics studies are featured as bulk analyses, which are based on a plenty of mixed cells. However, a tissue sample actually contains multiple types of cells. And even the gene expression profiles among morphologically indistinguishable cells are also highly heterogenous (70). As the emerging and developing of single-cell based technologies, it is possible to test the genome, transcriptome, proteome or metabolome considering cellular heterogeneity (71, 72). For example, the cell atlas of human placenta in GDM patients is constructed by single-cell RNA sequencing, and identifies cell type specific markers that may help to understand the molecular mechanism of GDM (73). In addition, the recent emerged spatial RNA sequencing can further map gene expressions to microstructural localizations by combining both histological imaging and RNA sequencing (74, 75). Similarly, spatial proteomics and metabolomics are also capable of detection of protein and metabolites spatially based on mass spectrometry imaging (76, 77).

### 6.3 New types of molecular targets

At transcript level, tRNA-derived small RNAs and circular RNAs have been the research hotspots and are reported to play important roles in various diseases (78, 79). For example, a circular RNA was found to be involved in the regulating of trophoblast functions in GDM (80). At protein level, more and more evidences have shown that novel small open reading frames (sORFs) are hidden in various non-coding transcripts (81). The sORF encoded small proteins can serve as a novel resource of disease markers. The canonical shot-gun proteomics strategy is mainly based on digested peptide fragments. However, various endogenous bioactive peptides are also known to be widely exist in human body with important biological functions (82). Thus, we suggest that both small proteins and bioactive peptides could be analyzed by peptidomics approach without digestion procedure. For example, over 200 peptides, which are found to be associated with gestational diabetes (GDM) induced fetal macrosomia, were identified in umbilical cord plasma (83). In addition to sequence cleavage, mature proteins may also be post-translationally modified at specific residues, which determine protein structure and functional activity. Various types of post-translational modifications (PTMs) are involved in almost all biological processes and highly associated with human diseases (84). However, there are few studies of protein PTMs in the field of perinatal complications.

### 6.4 From human sample to microbiota

Besides human genome, the metagenome of microbiota also plays important roles in regulating human health and disease (85). It was found that gut microbiome could be involved in the pathogenesis of pregnant conditions such as pre-eclampsia (PE) and gestational diabetes mellitus (GDM) (86, 87). There are also increasing evidences shown that maternal microbiota can influence fetal development and growth in recent years (88). Hence, metaomics approaches, especially metagenomics, have been the research hotspots in the field of perinatal medicine (89, 90). A recent mendelian randomization study indicated that there may exist causal association between gut microbiota and ICP (91). Thus, it will be interesting to investigate the role of microbiota in the occurrence and progression of ICP in the future. The mostly applied methods of studying microbiome are metagenome and 16S rRNA, which are based on the sequencing of the whole or specific genomic sequences (92). As the rapidly development of multiple omics technologies, it is also possible to perform other meta-omics analyses such as metatransciptomics, metaproteomics and meta-metabolomics (93).

### 6.5 From initial screening to the in-depth follow-up studies

Most of the studies are preliminary analyses based on the omics datasets, which only identify a list of potential key moleculars without in-depth investigation. Thus, it is needed to perform follow-up studies to further validate the results and to dig for more valuable information. According to the research objectives, there are also two directions for the follow-up studies: functional analysis and biomarkers analysis. For example, in the initial study of placental proteins between ICP and healthy pregnant women, a total of 38 differentially expressed (DE) proteins were identified (27). And the study only performed verification analysis on the expression of three apoptosis associated DE proteins (including ERp29, PRDX6 and MPO). However, in the following studies, the ERp29-centric regulation pathway and its downstream targets were comprehensively analyzed by using the cell models of ICP (94, 95).

Moreover, the published datasets provide valuable resources for the whole community to perform data mining and deep research by combining the recent novel findings in other fields. For example, several new cell death related pathways (including necroptosis, pyroptosis, ferroptosis, and cuproptosis) have been found to play important roles in various physiological and pathological processes (96). Among these pathways, ferroptosis is especially found to be associated with pregnancy related diseases and could be a potential target for therapy (97). As mentioned above, initial proteomics screening of placenta tissue showed that differentially expressed proteins are highly associated with apoptosis and autophagy based on functional annotation. However, bioinformatics annotation is relying on the knowledge-based database such as GO (Gene Ontology), which lacks of enough information about new findings. A recent study reanalyzed the published transcriptome and found that EGFR is the hub gene associated with destroyed autophagy and ferroptosis in placenta (98). Hence, it is reasonable and necessary to carry out more additional studies to verify if these new cell death related pathway are involved in the pathogenesis of ICP.

### 6.6 From single omics to integrated multi-omics studies

Furthermore, the integration of multiple omics datasets could provide a multifaceted and holistic overview of the research content (99, 100). Here, we broadly classify the integration studies into three different levels. Firstly, the integration of different batches can be used to increase sample size. For example, in the genome-wide association studies (GWAS) analysis of ICP, a total of three large cohorts (including 1138 cases and 153,642 controls) were combined in order to obtain a large sample of ICP (14). Secondly, the integration of different subtype of moleculars is useful for a more comprehensive analysis. For example, the transcripts contain multiple subtypes including mRNA, lncRNA and microRNA. By combining these three subtypes of RNAs, a competing endogenous RNA (ceRNA) network was constructed to infer the regulatory relations and pathways involved in the pathogenesis of ICP (32). Thirdly, the integration of different types of moleculars can be performed for a more systematic analysis. For example, by combining proteomics and metabolomics data, the key protein (Peroxisomal ACOX1) and its substrate (L-palmitoylcarnitine) were found to be both differentially expressed and involved in fatty acid metabolism (45).

## 7 Conclusions

Conclusively, ICP is a self-limiting hepatic disease during the perinatal period. However, it could be a threat to both maternal and fetal health. Recent studies also showed that ICP might have long-term effects on both the pregnant women and the child. Hence, it is of vital importance to identify key moleculars and the associated functional pathways of ICP, which can help us to better understand the pathological mechanisms as well as to develop precise clinical biomarkers. Over the past decades, the emerging and developing of various omics methods offer large-scale approaches to systematically study the genome, transcriptome, proteome and metabolome of clinical samples. In the present review, we comprehensively collected and summarized the studies of ICP based on genomics, transcriptomics, proteomics, and metabolomics strategies. In brief, the experimental designs of these studies include different research objects (such as ICP, ICP subtypes, ICP fetuses, ICP models, and other complications). The studied samples contain clinical samples (including placenta, blood, serum, plasma, urine, and hair), *in vitro* cultured tissues, cell lines and the samples from animal models. According to the main research objectives, we further categorized these studies into two groups: pathogenesis and diagnosis analyses. The pathogenesis studies identified tens of functional pathways that may represent the key regulatory events for the occurrence, progression, treatment and fetal effects of ICP. On the other hand, the diagnosis studies provide more than 40 potential models for the early-prediction, diagnosis, grading, prognosis or differential diagnosis of ICP. Additionally, we also evaluated the limitations of current studies by following the suggestions proposed in a previous framework of precision and translational proteomics. Specifically, we emphasized that many aspects of clinical characteristics, sample processing, and analytical method can greatly affect the reliability and repeatability of omics results. We also propose to share the omics datasets for the community to perform reanalysis and in-depth follow-up studies. Finally, although these omics studies have already covered a broad range of research contents, there are still many new research topics that needed to be addressed in the future. We thus pointed out 6 new research directions including new clinical issues, new strategies, new molecular types, microbiata, follow-up studies and integrated studies, which may benefit the omics based analyses of ICP and other perinatal associated conditions in the future.

## Author contributions

MW: Writing – review & editing, Investigation, Writing – original draft. LC: Resources, Writing – original draft, Writing – review & editing. JLi: Visualization, Writing – original draft, Writing – review & editing. YY: Data curation, Resources, Writing – review & editing. ZQ: Data curation, Resources, Writing – review & editing. JLiu: Data curation, Resources, Writing – review & editing. YJ: Data curation, Resources, Writing – review & editing. TZ: Conceptualization, Methodology, Supervision, Writing – review & editing. YG: Conceptualization, Methodology, Supervision, Writing – review & editing. YZ: Conceptualization, Funding acquisition, Methodology, Supervision, Writing – review & editing.

## References

[1] Smith DD Rood KM. Intrahepatic cholestasis of pregnancy. Clin Obstet Gynecol (2020) 63(1):134–51. doi: 10.1097/grf.0000000000000495 31764000

[2] Gao XX Ye MY Liu Y Li JY Li L Chen W. Prevalence and risk factors of intrahepatic cholestasis of pregnancy in a Chinese population. Sci Rep (2020) 10(1):16307. doi: 10.1038/s41598-020-73378-5 33004915 PMC7530728

[3] Piechota J Jelski W. Intrahepatic cholestasis in pregnancy: review of the literature. J Clin Med (2020) 9(5):7. doi: 10.3390/jcm9051361 PMC7290322 32384779

[4] Hagenbeck C Hamza A Kehl S Maul H Lammert F Keitel V. Management of intrahepatic cholestasis of pregnancy: recommendations of the working group on obstetrics and prenatal medicine - section on maternal disorders. Geburtshilfe und Frauenheilkunde (2021) 81(8):922–39. doi: 10.1055/a-1386-3912 PMC8354365 34393256

[5] Arafa A Dong JY. Association between intrahepatic cholestasis of pregnancy and risk of gestational diabetes and preeclampsia: a systematic review and meta-analysis. Hypertens Pregnancy (2020) 39(3):354–60. doi: 10.1080/10641955.2020.1758939 32326772

[6] Zhang L Tang C Ye C Huang L Wu Y. Intrahepatic cholestasis of pregnancy can increase the risk of metabolic disorders: A meta-analysis. J Med Biochem (2022) 41(4):549–58. doi: 10.5937/jomb0-33222 PMC9618343 36381082

[7] Diken Z Usta IM Nassar AH. A clinical approach to intrahepatic cholestasis of pregnancy. Am J Perinatol (2014) 31(1):1–8. doi: 10.1055/s-0033-1333673 23359238

[8] Arrese M Macias RI Briz O Perez MJ Marin JJ. Molecular pathogenesis of intrahepatic cholestasis of pregnancy. Expert Rev Mol Med (2008) 10:e9. doi: 10.1017/s1462399408000628 18371245

[9] Lammert F Marschall HU Glantz A Matern S. Intrahepatic cholestasis of pregnancy: molecular pathogenesis, diagnosis and management. J Hepatol (2000) 33(6):1012–21. doi: 10.1016/s0168-8278(00)80139-7 11131439

[10] Ozen M Aghaeepour N Marić I Wong RJ Stevenson DK Jantzie LL. Omics approaches: interactions at the maternal-fetal interface and origins of child health and disease. Pediatr Res (2023) 93(2):366–75. doi: 10.1038/s41390-022-02335-x PMC9549444 36216868

[11] Wood AM Livingston EG Hughes BL Kuller JA. Intrahepatic cholestasis of pregnancy: A review of diagnosis and management. Obstetrical Gynecological Survey (2018) 73(2):103–9. doi: 10.1097/ogx.0000000000000524 29480924

[12] Walker KF Chappell LC Hague WM Middleton P Thornton JG. Pharmacological interventions for treating intrahepatic cholestasis of pregnancy. Cochrane Database Sys Rev (2020) 7(7):CD000493. doi: 10.1002/14651858.CD000493.pub3 PMC7389072 32716060

[13] Xiao J Li Z Song Y Sun Y Shi H Chen D. Molecular pathogenesis of intrahepatic cholestasis of pregnancy. Can J Gastroenterol Hepatol (2021) 2021:6679322. doi: 10.1155/2021/6679322 34195157 PMC8181114

[14] Dixon PH Levine AP Cebola I Chan MMY Amin AS Aich A. GWAS meta-analysis of intrahepatic cholestasis of pregnancy implicates multiple hepatic genes and regulatory elements. Nat Commun (2022) 13(1):4840. doi: 10.1038/s41467-022-29931-z 35977952 PMC9385867

[15] Li B Ritchie MD. From GWAS to gene: transcriptome-wide association studies and other methods to functionally understand GWAS discoveries. Front Genet (2021) 12:713230. doi: 10.3389/fgene.2021.713230 34659337 PMC8515949

[16] Cui T Liu Y Men X Xu Z Wu L Liu S. Bile acid transport correlative protein mRNA expression profile in human placenta with intrahepatic cholestasis of pregnancy. Saudi Med J (2009) 30(11):1406–10. 19882051

[17] Wei J Wang H Yang X Dong M Wang Z. Altered gene profile of placenta from women with intrahepatic cholestasis of pregnancy. Arch Gynecol Obstet (2010) 281(5):801–10. doi: 10.1007/s00404-009-1156-3 19565256

[18] Milona A Owen BM Cobbold JF Willemsen EC Cox IJ Boudjelal M. Raised hepatic bile acid concentrations during pregnancy in mice are associated with reduced farnesoid X receptor function. Hepatol (Baltimore Md) (2010) 52(4):1341–9. doi: 10.1002/hep.23849 20842631

[19] Papacleovoulou G Abu-Hayyeh S Nikolopoulou E Briz O Owen BM Nikolova V. Maternal cholestasis during pregnancy programs metabolic disease in offspring. J Clin Invest (2013) 123(7):3172–81. doi: 10.1172/jci68927 PMC3696570 23934127

[20] Du Q Pan Y Zhang Y Zhang H Zheng Y Lu L. Placental gene-expression profiles of intrahepatic cholestasis of pregnancy reveal involvement of multiple molecular pathways in blood vessel formation and inflammation. BMC Med Genomics (2014) 7:42. doi: 10.1186/1755-8794-7-42 25001852 PMC4105836

[21] Mella MT Kohari K Jones R Peña J Ferrara L Stone J. Mitochondrial gene expression profiles are associated with intrahepatic cholestasis of pregnancy. Placenta (2016) 45:16–23. doi: 10.1016/j.placenta.2016.07.002 27577705

[22] Zou P Luo L Zhao C Chen Z Dong R Li N. The serum microRNA profile of intrahepatic cholestasis of pregnancy: identification of novel noninvasive biomarkers. Cell Physiol Biochem (2018) 51(3):1480–8. doi: 10.1159/000495595 30485846

[23] Zou S Zhao S Wang J Dong R Zou P Liang F. Diagnostic and prognostic value of long noncoding RNAs as potential novel biomarkers in intrahepatic cholestasis of pregnancy. BioMed Res Int (2021) 2021:8858326. doi: 10.1155/2021/8858326 33728343 PMC7936904

[24] Zu Y Guo S Li G Gao Q Wang X Zhang C. Serum microRNAs as non-invasive diagnostic biomarkers for intrahepatic cholestasis of pregnancy. Am J Trans Res (2022) 14(9):6763–73. PMC9556493 36247288

[25] Zhang W Yu Y Hertwig F Thierry-Mieg J Thierry-Mieg D Wang J. Comparison of RNA-seq and microarray-based models for clinical endpoint prediction. Genome Biol (2015) 16(1):133. doi: 10.1186/s13059-015-0694-1 26109056 PMC4506430

[26] Yeung ES. Genome-wide correlation between mRNA and protein in a single cell. Angewandte Chemie (International ed English) (2011) 50(3):583–5. doi: 10.1002/anie.201005969 21226136

[27] Zhang T Guo Y Guo X Zhou T Chen D Xiang J. Comparative proteomics analysis of placenta from pregnant women with intrahepatic cholestasis of pregnancy. PloS One (2013) 8(12):e83281. doi: 10.1371/journal.pone.0083281 24391750 PMC3877025

[28] He P Wang F Jiang Y Zhong Y Lan Y Chen S. Placental proteome alterations in women with intrahepatic cholestasis of pregnancy. Int J Gynaecol Obstet (2014) 126(3):256–9. doi: 10.1016/j.ijgo.2014.03.035 24913202

[29] Lofthouse EM Torrens C Manousopoulou A Nahar M Cleal JK O’Kelly IM. Ursodeoxycholic acid inhibits uptake and vasoconstrictor effects of taurocholate in human placenta. FASEB J (2019) 33(7):8211–20. doi: 10.1096/fj.201900015RR PMC6593889 30922127

[30] Chao S Xiaojun L Haizhen W Ludi F Shaozhen L Zhiwen S. Lithocholic acid activates mTOR signaling inducing endoplasmic reticulum stress in placenta during intrahepatic cholestasis of pregnancy. Life Sci (2019) 218:300–7. doi: 10.1016/j.lfs.2018.12.050 30605648

[31] Zou S Dong R Wang J Liang F Zhu T Zhao S. Use of data-independent acquisition mass spectrometry for comparative proteomics analyses of sera from pregnant women with intrahepatic cholestasis of pregnancy. J Proteomics (2021) 236:104124. doi: 10.1016/j.jprot.2021.104124 33545297

[32] Fang D Fang Y Zhang W Xiang Y Cheng X Liang M. Comprehensive analysis of quantitative proteomics with DIA mass spectrometry and ceRNA network in intrahepatic cholestasis of pregnancy. Front Cell Dev Biol (2022) 10:854425. doi: 10.3389/fcell.2022.854425 35938169 PMC9354660

[33] Yang X Zhou Y Li H Song F Li J Zhang Y. Autophagic flux inhibition, apoptosis, and mitochondrial dysfunction in bile acids-induced impairment of human placental trophoblast. J Cell Physiol (2022) 237(7):3080–94. doi: 10.1002/jcp.30774 35579960

[34] Jiang Y Yin X Xu Q Tang X Zhang H Cao X. SWATH proteomics analysis of placental tissue with intrahepatic cholestasis of pregnancy. Placenta (2023) 137:1–13. doi: 10.1016/j.placenta.2023.04.009 37054625

[35] Nie L Xin S Zheng J Luo Y Zou Y Liu X. DIA-based proteomics analysis of serum-derived exosomal proteins as potential candidate biomarkers for intrahepatic cholestasis in pregnancy. Arch Gynecol Obstet (2022) 308(1):79–11. doi: 10.1007/s00404-022-06703-0 35849169

[36] Cui M Cheng C Zhang L. High-throughput proteomics: a methodological mini-review. Lab Invest (2022) 102(11):1170–81. doi: 10.1038/s41374-022-00830-7 PMC9362039 35922478

[37] Costa Dos Santos G Jr. Renovato-Martins M de Brito NM. The remodel of the “central dogma”: a metabolomics interaction perspective. Metabolomics (2021) 17(5):48. doi: 10.1007/s11306-021-01800-8 33969452 PMC8106972

[38] Gao J Xu B Zhang X Cui Y Deng L Shi Z. Association between serum bile acid profiles and gestational diabetes mellitus: A targeted metabolomics study. Clin Chim Acta (2016) 459:63–72. doi: 10.1016/j.cca.2016.05.026 27246871

[39] Ma L Zhang X Pan F Cui Y Yang T Deng L. Urinary metabolomic analysis of intrahepatic cholestasis of pregnancy based on high performance liquid chromatography/mass spectrometry. Clin Chim Acta (2017) 471:292–7. doi: 10.1016/j.cca.2017.06.021 28669684

[40] de Seymour JV Tu S He X Zhang H Han TL Baker PN. Metabolomic profiling of maternal hair suggests rapid development of intrahepatic cholestasis of pregnancy. Metabolomics (2018) 14(6):79. doi: 10.1007/s11306-018-1371-7 30830343

[41] Li Y Zhang X Chen J Feng C He Y Shao Y. Targeted metabolomics of sulfated bile acids in urine for the diagnosis and grading of intrahepatic cholestasis of pregnancy. Genes Dis (2018) 5(4):358–66. doi: 10.1016/j.gendis.2018.01.005 PMC6304334 30591938

[42] Cui Y Xu B Zhang X He Y Shao Y Ding M. Diagnostic and therapeutic profiles of serum bile acids in women with intrahepatic cholestasis of pregnancy-a pseudo-targeted metabolomics study. Clin Chim Acta (2018) 483:135–41. doi: 10.1016/j.cca.2018.04.035 29709452

[43] Chen X Zhang X Xu B Cui Y He Y Yang T. The urinary bile acid profiling analysis of asymptomatic hypercholanemia of pregnancy: A pseudo-targeted metabolomics study. Clin Chim Acta (2019) 497:67–75. doi: 10.1016/j.cca.2019.07.002 31276634

[44] Wang P Zhong H Song Y Yuan P Li Y Lin S. Targeted metabolomics analysis of maternal-placental-fetal metabolism in pregnant swine reveals links in fetal bile acid homeostasis and sulfation capacity. Am J Physiol Gastrointestinal Liver Physiol (2019) 317(1):G8–G16. doi: 10.1152/ajpgi.00056.2019 31021171

[45] Dong R Ye N Zhao S Wang G Zhang Y Wang T. Studies on novel diagnostic and predictive biomarkers of intrahepatic cholestasis of pregnancy through metabolomics and proteomics. Front Immunol (2021) 12:733225. doi: 10.3389/fimmu.2021.733225 34721396 PMC8552060

[46] Zheng Q Shen L Zhao D Zhang H Liang Y Zhu Y. Metabolic characteristics of plasma bile acids in patients with intrahepatic cholestasis of pregnancy-mass spectrometric study. Metabolomics (2021) 17(10):93. doi: 10.1007/s11306-021-01844-w 34595616

[47] Shao Y Chen S Li H Tang Q Xu D. Maternal bile acid profile and subtype analysis of intrahepatic cholestasis of pregnancy. Orphanet J Rare Dis (2021) 16(1):259. doi: 10.1186/s13023-021-01887-1 34098996 PMC8186144

[48] Wang J Wen J Ma X Yang J Zhang Z Xie S. Validation of MAPK signalling pathway as a key role of paeoniflorin in the treatment of intrahepatic cholestasis of pregnancy based on network pharmacology and metabolomics. Eur J Pharmacol (2022) 935:175331. doi: 10.1016/j.ejphar.2022.175331 36273619

[49] Liu W Wang Q Chang J Bhetuwal A Bhattarai N Ni X. Circulatory metabolomics reveals the association of the metabolites with clinical features in the patients with intrahepatic cholestasis of pregnancy. Front Physiol (2022) 13:848508. doi: 10.3389/fphys.2022.848508 35899031 PMC9309339

[50] Sun X Qu T Wang W Li C Yang X He X. Untargeted lipidomics analysis in women with intrahepatic cholestasis of pregnancy: a cross-sectional study. BJOG: an Int J Obstet Gynaecol (2022) 129(6):880–8. doi: 10.1111/1471-0528.17026 34797934

[51] He Y Zhang X Shao Y Xu B Cui Y Chen X. Recognition of asymptomatic hypercholanemia of pregnancy: Different clinical features, fetal outcomes and bile acids metabolism from intrahepatic cholestasis of pregnancy. Biochim Biophys Acta Mol Basis Dis (2022) 1868(1):166269. doi: 10.1016/j.bbadis.2021.166269 34537368

[52] Alaei Faradonbeh F Lastuvkova H Cermanova J Hroch M Nova Z Uher M. Multidrug resistance-associated protein 2 deficiency aggravates estrogen-induced impairment of bile acid metabolomics in rats. Front Physiol (2022) 13:859294. doi: 10.3389/fphys.2022.859294 35388287 PMC8979289

[53] Xu H Xu Y Zhao G Fu X Zhao J Wang H. The complete change in bile acids and steroids in systematic metabolomics applied to the intrahepatic cholestasis of pregnancy. Mol Omics (2023) 19(5):418–11. doi: 10.1039/d2mo00305h 37000693

[54] Heiles S. Advanced tandem mass spectrometry in metabolomics and lipidomics-methods and applications. Anal Bioanal Chem (2021) 413(24):5927–48. doi: 10.1007/s00216-021-03425-1 PMC8440309 34142202

[55] Zeki ÖC Eylem CC Reçber T Kır S Nemutlu E. Integration of GC-MS and LC-MS for untargeted metabolomics profiling. J Pharm Biomed Anal (2020) 190:113509. doi: 10.1016/j.jpba.2020.113509 32814263

[56] Xu L Wu LF Deng FY. Exosome: an emerging source of biomarkers for human diseases. Curr Mol Med (2019) 19(6):387–94. doi: 10.2174/1566524019666190429144310 31288712

[57] Wang M Chen D Xia Y Zhou T Jiang S-W. A refined framework for precision and translational proteomics in clinical research. Curr Proteomics (2021) 18(4):436–46. doi: 10.2174/1570164617999201110122901

[58] Mayhew TM. Taking tissue samples from the placenta: an illustration of principles and strategies. Placenta (2008) 29(1):1–14. doi: 10.1016/j.placenta.2007.05.010 17658596

[59] Meyer JG Schilling B. Clinical applications of quantitative proteomics using targeted and untargeted data-independent acquisition techniques. Expert Rev Proteomics (2017) 14(5):419–29. doi: 10.1080/14789450.2017.1322904 PMC5671767 28436239

[60] Shumway M Cochrane G Sugawara H. Archiving next generation sequencing data. Nucleic Acids Res (2010) 38(Database issue):D870–1. doi: 10.1093/nar/gkp1078 PMC2808927 19965774

[61] Hermjakob H Apweiler R. The Proteomics Identifications Database (PRIDE) and the ProteomExchange Consortium: making proteomics data accessible. Expert Rev Proteomics (2006) 3(1):1–3. doi: 10.1586/14789450.3.1.1 16445344

[62] Kale NS Haug K Conesa P Jayseelan K Moreno P Rocca-Serra P. MetaboLights: an open-access database repository for metabolomics data. Curr Protoc Bioinf (2016) 53:14 3 1– 3 8. doi: 10.1002/0471250953.bi1413s53 27010336

[63] Dunkler D Sánchez-Cabo F Heinze G. Statistical analysis principles for Omics data. Methods Mol Biol (Clifton NJ) (2011) 719:113–31. doi: 10.1007/978-1-61779-027-0_5 21370081

[64] Badon SE Zhu Y Sridhar SB Xu F Lee C Ehrlich SF. A pre-pregnancy biomarker risk score improves prediction of future gestational diabetes. J Endocr Soc (2018) 2(10):1158–69. doi: 10.1210/js.2018-00200 PMC6169465 30302420

[65] Bicocca MJ Sperling JD Chauhan SP. Intrahepatic cholestasis of pregnancy: Review of six national and regional guidelines. Eur J Obstet Gynecol Reprod Biol (2018) 231:180–7. doi: 10.1016/j.ejogrb.2018.10.041 30396107

[66] Lin J Gu W Hou Y. Diagnosis and prognosis of early-onset intrahepatic cholestasis of pregnancy: a prospective study. J Maternal-fetal Neonatal Med (2019) 32(6):997–1003. doi: 10.1080/14767058.2017.1397124 29065754

[67] Bacq Y le Besco M Lecuyer AI Gendrot C Potin J Andres CR. Ursodeoxycholic acid therapy in intrahepatic cholestasis of pregnancy: Results in real-world conditions and factors predictive of response to treatment. Digestive Liver Dis (2017) 49(1):63–9. doi: 10.1016/j.dld.2016.10.006 27825922

[68] Grady J Clifford C Treadwell MC Parikh ND Satishchandran A. Use of fenofibrate for intrahepatic cholestasis of pregnancy. J Hepatol (2023) 79(2):84–3. doi: 10.1016/j.jhep.2023.04.014 37084798

[69] Geenes V Chambers J Khurana R Shemer EW Sia W Mandair D. Rifampicin in the treatment of severe intrahepatic cholestasis of pregnancy. Eur J Obstet Gynecol Reprod Biol (2015) 189:59–63. doi: 10.1016/j.ejogrb.2015.03.020 25864112

[70] Carter B Zhao K. The epigenetic basis of cellular heterogeneity. Nat Rev Genet (2021) 22(4):235–50. doi: 10.1038/s41576-020-00300-0 PMC10880028 33244170

[71] Stein CM Weiskirchen R Damm F Strzelecka PM. Single-cell omics: Overview, analysis, and application in biomedical science. J Cell Biochem (2021) 122(11):1571–8. doi: 10.1002/jcb.30134 34459502

[72] Baysoy A Bai Z Satija R Fan R. The technological landscape and applications of single-cell multi-omics. Nat Rev Mol Cell Biol (2023) 24(10):695–19. doi: 10.1038/s41580-023-00615-w PMC10242609 37280296

[73] Yang Y Guo F Peng Y Chen R Zhou W Wang H. Transcriptomic profiling of human placenta in gestational diabetes mellitus at the single-cell level. Front Endocrinol (2021) 12:679582. doi: 10.3389/fendo.2021.679582 PMC8139321 34025588

[74] Williams CG Lee HJ Asatsuma T Vento-Tormo R Haque A. An introduction to spatial transcriptomics for biomedical research. Genome Med (2022) 14(1):68. doi: 10.1186/s13073-022-01075-1 35761361 PMC9238181

[75] Vandereyken K Sifrim A Thienpont B Voet T. Methods and applications for single-cell and spatial multi-omics. Nat Rev Genet (2023) 24(8):494–22. doi: 10.1038/s41576-023-00580-2 PMC9979144 36864178

[76] Guo G Papanicolaou M Demarais NJ Wang Z Schey KL Timpson P. Automated annotation and visualisation of high-resolution spatial proteomic mass spectrometry imaging data using HIT-MAP. Nat Commun (2021) 12(1):3241. doi: 10.1038/s41467-021-23461-w 34050164 PMC8163805

[77] Alexandrov T. Spatial metabolomics and imaging mass spectrometry in the age of artificial intelligence. Annu Rev Biomed Data Sci (2020) 3:61–87. doi: 10.1146/annurev-biodatasci-011420-031537 34056560 PMC7610844

[78] Liu B Cao J Wang X Guo C Liu Y Wang T. Deciphering the tRNA-derived small RNAs: origin, development, and future. Cell Death Dis (2021) 13(1):24. doi: 10.1038/s41419-021-04472-3 34934044 PMC8692627

[79] Wang CC Han CD Zhao Q Chen X. Circular RNAs and complex diseases: from experimental results to computational models. Briefings Bioinf (2021) 22(6):27. doi: 10.1093/bib/bbab286 PMC8575014 34329377

[80] Chen H Zhang S Wu Y Li Z Wang D Cai S. The role of circular RNA circ_0008285 in gestational diabetes mellitus by regulating the biological functions of trophoblasts. Biol Res (2021) 54(1):14. doi: 10.1186/s40659-021-00337-3 33879262 PMC8056579

[81] Liu H Zhou X Yuan M Zhou S Huang YE Hou F. ncEP: A manually curated database for experimentally validated ncRNA-encoded proteins or peptides. J Mol Biol (2020) 432(11):3364–8. doi: 10.1016/j.jmb.2020.02.022 32105730

[82] Foreman RE George AL Reimann F Gribble FM Kay RG. Peptidomics: A review of clinical applications and methodologies. J Proteome Res (2021) 20(8):3782–97. doi: 10.1021/acs.jproteome.1c00295 34270237

[83] Liu F Zhao C Liu L Ding H Huo R Shi Z. Peptidome profiling of umbilical cord plasma associated with gestational diabetes-induced fetal macrosomia. J Proteomics (2016) 139:38–44. doi: 10.1016/j.jprot.2016.03.001 26945739

[84] Xu H Wang Y Lin S Deng W Peng D Cui Q. PTMD: A database of human disease-associated post-translational modifications. Genomics Proteomics Bioinf (2018) 16(4):244–51. doi: 10.1016/j.gpb.2018.06.004 PMC6205080 30244175

[85] Brüls T Weissenbach J. The human metagenome: our other genome? Hum Mol Genet (2011) 20(R2):R142–8. doi: 10.1093/hmg/ddr353 21840927

[86] Chen X Li P Liu M Zheng H He Y Chen MX. Gut dysbiosis induces the development of pre-eclampsia through bacterial translocation. Gut (2020) 69(3):513–22. doi: 10.1136/gutjnl-2019-319101 31900289

[87] Hasain Z Mokhtar NM Kamaruddin NA Mohamed Ismail NA Razalli NH Gnanou JV. Gut microbiota and gestational diabetes mellitus: A review of host-gut microbiota interactions and their therapeutic potential. Front Cell Infect Microbiol (2020) 10:188. doi: 10.3389/fcimb.2020.00188 32500037 PMC7243459

[88] Yao Y Cai X Chen C Fang H Zhao Y Fei W. The role of microbiomes in pregnant women and offspring: research progress of recent years. Front Pharmacol (2020) 11:643. doi: 10.3389/fphar.2020.00643 32457628 PMC7225329

[89] Wang L Li F Gu B Qu P Liu Q Wang J. Metaomics in clinical laboratory: potential driving force for innovative disease diagnosis. Front Microbiol (2022) 13:883734. doi: 10.3389/fmicb.2022.883734 35783436 PMC9247514

[90] Fox C Eichelberger K. Maternal microbiome and pregnancy outcomes. Fertil Steril (2015) 104(6):1358–63. doi: 10.1016/j.fertnstert.2015.09.037 26493119

[91] Li C Li N Liu C Yin S. Causal association between gut microbiota and intrahepatic cholestasis of pregnancy: mendelian randomization study. BMC Pregnancy Childbirth (2023) 23(1):568. doi: 10.1186/s12884-023-05889-8 37543573 PMC10403878

[92] Durazzi F Sala C Castellani G Manfreda G Remondini D De Cesare A. Comparison between 16S rRNA and shotgun sequencing data for the taxonomic characterization of the gut microbiota. Sci Rep (2021) 11(1):3030. doi: 10.1038/s41598-021-82726-y 33542369 PMC7862389

[93] Zhang X Li L Butcher J Stintzi A Figeys D. Advancing functional and translational microbiome research using meta-omics approaches. Microbiome (2019) 7(1):154. doi: 10.1186/s40168-019-0767-6 31810497 PMC6898977

[94] Zhang T Zhao C Luo L Xiang J Cheng J Wang T. High concentraction of taurocholic acid induced apoptosis in HTR-8/SVneo cells via overexpression of ERp29 and activation of p38. Placenta (2014) 35(7):496–500. doi: 10.1016/j.placenta.2014.03.023 24780196

[95] Zou S Zou P Wang Y Dong R Wang J Li N. ERp29 inhibition attenuates TCA toxicity via affecting p38/p53- dependent pathway in human trophoblast HTR-8/SVeno cells. Arch Biochem Biophys (2019) 676:108125. doi: 10.1016/j.abb.2019.108125 31586554

[96] Xue Q Kang R Klionsky DJ Tang D Liu J Chen X. Copper metabolism in cell death and autophagy. Autophagy (2023) 19(8):2175–95. doi: 10.1080/15548627.2023.2200554 PMC10351475 37055935

[97] Xu J Zhou F Wang X Mo C. Role of ferroptosis in pregnancy related diseases and its therapeutic potential. Front Cell Dev Biol (2023) 11:1083838. doi: 10.3389/fcell.2023.1083838 36968201 PMC10031498

[98] Fang Y Fang D. Comprehensive analysis of placental gene-expression profiles and identification of EGFR-mediated autophagy and ferroptosis suppression in intrahepatic cholestasis of pregnancy. Gene (2022) 834:146594. doi: 10.1016/j.gene.2022.146594 35643225

[99] Miao Z Humphreys BD McMahon AP Kim J. Multi-omics integration in the age of million single-cell data. Nat Rev Nephrol (2021) 17(11):710–24. doi: 10.1038/s41581-021-00463-x PMC9191639 34417589

[100] Subramanian I Verma S Kumar S Jere A Anamika K. Multi-omics data integration, interpretation, and its application. Bioinf Biol Insights (2020) 14:1177932219899051. doi: 10.1177/1177932219899051 PMC7003173 32076369

